# Effects of the Ultrasonic Assisted Surface Rolling Process on the Fatigue Crack Initiation Position Distribution and Fatigue Life of 51CrV4 Spring Steel

**DOI:** 10.3390/ma14102565

**Published:** 2021-05-14

**Authors:** Changxing Xu, Yilong Liang, Ming Yang, Jiabang Yu, Xiang Peng

**Affiliations:** 1 College of Materials and Metallurgy, Guizhou University, Guiyang 550025, China; xuchangxing7@126.com (C.X.); myang5@gzu.edu.cn (M.Y.); yujiabang1314@126.com (J.Y.); pengxiang220@126.com (X.P.); 2 Key Laboratory for Mechanical Behavior and Microstructure of Materials of Guizhou Province, Guiyang 550025, China; 3 National & Local Joint Engineering Laboratory for High-performance Metal Structure Material and Advanced Manufacturing Technology, Guiyang 550025, China

**Keywords:** ultrasonic assisted surface rolling process, 51CrV4 spring steel, modified gradient field, fatigue crack initiation position, stress triaxiality

## Abstract

In this paper, the effects of the fatigue crack initiation position (FCIP) on fatigue life are discussed. Different modified gradient fields (MGFs) are prepared on the surface of 51CrV4 spring steel components by an ultrasonic assisted surface rolling process (USRP). Subsequently, the fatigue behaviour of steels with different FCIPs is systematically studied. The results show that the fatigue life of steels first exhibits an increasing tendency and then a decreasing tendency with increasing distance between an FCIP and the surface. When an FCIP shifts from the surface of the sample to the interior, the fatigue crack initiation resistance on the interior is greater than that on the surface, which leads to an increase in fatigue life. However, when the FCIP further shifts towards the centre of the specimen, the stress triaxiality experienced by the fatigue source gradually increases, which results in a peak in the curve of FCIP versus fatigue life. The magnitude of this peak fatigue life is related to the change in the stress triaxiality. Moreover, according to focused ion beam-high-resolution transmission electron microscopy (FIB-HRTEM) microstructural analysis near FCIPs, under a higher stress triaxiality, the crack tip area is subject to greater stress constraints, making the multiplication and movement of dislocations in this area more difficult, resulting in the decrease in movable dislocation density. This decrease in dislocation density leads to an increase in the stress concentration and accelerates the crack growth rate, decreasing the fatigue life. Therefore, the significant change in fatigue life is controlled by the MGF and stress triaxiality.

## 1. Introduction

As the material of high-speed train bogies, 51CrV4 spring steel has the advantages of high strength, high fatigue resistance and low overheating sensitivity. Therefore, 51CrV4 spring steel is employed in parts used for bearing, buffering and steering during operation. However, during long-term operation, new defects and cracks will be formed on the surface of the material, which will promote the initiation of fatigue cracks, which will lead to a decrease in fatigue life [1,2]. According to related research reports [3,4], with high-energy surface strengthening treatment, the surface integrity of the material can be improved, which lengthens fatigue life. Therefore, high-energy surface strengthening technology is generally used to improve the fatigue life of key components. Commonly used technologies for high-energy surface strengthening mainly include abrasive water jet peening (AWJP) [5,6], laser shock processing (LSP) [7,8], ultrasonic shot peening (USP) [9,10] and the ultrasonic-assisted surface rolling process (USRP) [11,12]. Compared with other high-energy surface strengthening technologies, the USRP can greatly improve the surface roughness of a specimen and eliminate the processing traces caused by cold working. In addition, the USRP can not only form a nanoscale microstructure gradient on the surface of the material but also significantly improve the microhardness gradient and residual stress gradient on the surface of the material. However, these effects cannot be achieved by traditional surface strengthening technology [13,14].

Several researchers [15,16,17] have reported that after high-energy surface strengthening treatment of a specimen, the compressive residual stress layer (CRSL) formed tends to shift the fatigue crack initiation position (FCIP) from the specimen’s surface to its interior, thereby increasing the fatigue life. For instance, after surface spinning strengthening (3S) treatment on a rotating bending specimen of 50CrMnMoVNb spring steel (the minimum diameter of the specimen was ϕ4 mm), Ren et al. [18] found that the surface decarburized spring steel was treated by 3S, the FCIP migrated to the subsurface layer (i.e., 0 ~225 μm) and the fatigue life was improved. After the surface of spring steel was strengthened by 3S, the grains in the surface layer were refined, thereby resulting in work hardening, and the ultimate tensile strength of this surficial work hardened layer was increased. This makes it difficult for fatigue cracks to initiate in the work-hardened layer, and thus can lead to an increase in fatigue life. In addition, the internal FCIPs of the spring steel with fatigue failure were all triggered by inclusions, and the size of the inclusions was positively correlated with the increase in fatigue life. After USRP treatment on a rotating bending specimen of Ti-6Al-4V alloy (the minimum diameter of the specimen was ϕ6 mm), Liu et al. [19] found that FCIP of each sample gradually moved to the inside with fewer rolling times (i.e., 748~870 μm), thereby increasing the fatigue life of the alloy. With decreasing processing times, the lower the surface roughness of the specimen was, the greater the value of the surface compressive residual stress. Therefore, an improvement in the surface integrity of a material can prevent the initiation and premature propagation of fatigue cracks, thus slowing the crack growth rate and increasing the fatigue life. For high-energy surface modification, among the factors influencing the improvement in fatigue performance reported above, only the influence of the surface integrity of the specimen and the size of the inclusions on fatigue life have been considered, not the influence of the FCIP on the fatigue life. Wang [20] suggested that the restraining force of the dislocation reciprocating motion caused by the initiation of internal fatigue cracks in a material is different from that of the surface, namely, the fatigue life of the internal FCIP is 1.35~1.4 times that of the surface. In other words, when the FCIP shifts from the surface of the specimen to the interior, the fatigue life will be improved. However, this theory considers only the difference in fatigue crack initiation resistance between the surface and interior of the specimen, not the influence of the FCIP close to the centre of the specimen, on the fatigue life, and research on this behaviour is still lacking.

Based on the background information presented above, this study uses USRP technology to generate different modified gradient fields (MGFs) on 51CrV4 spring steel specimens to induce different FCIPs. Then, the relationship between the FCIPs and the fatigue life is observed, and the microstructural characteristics of different fatigue crack tips are analysed to clarify the influence mechanism of different MGFs on fatigue life.

## 2. Experimental

### 2.1. Materials

Industrially annealed 51CrV4 spring steel rods were processed into cylindrical specimens of ϕ30 mm × 150 mm, and then three-step heat treatment experiments were carried out on the material: (1) normalising: 950 °C × 20 min + air cooling; (2) quenching: 835 °C × 20 min + 15% polyaleneglycol (PAG) quenching; and (3) tempering: 400 °C × 90 min + air cooling. The chemical composition of 51CrV4 spring steel before heat treatment is shown in Table 1, and the tensile properties after heat treatment are shown in Table 2.

### 2.2. Fatigue Testing

**Figure 1:**
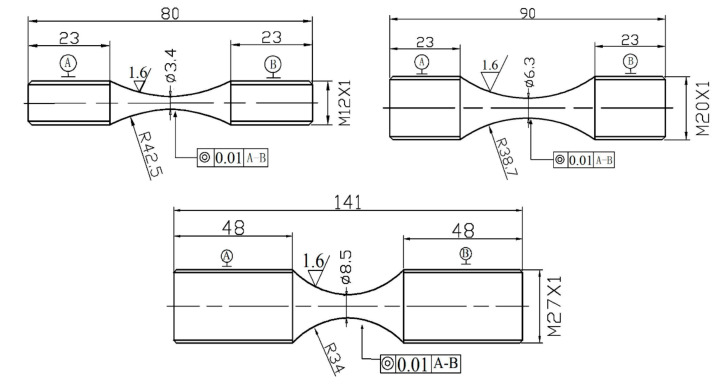
Geometry of fatigue test specimens (units: mm).

**Figure 2:**
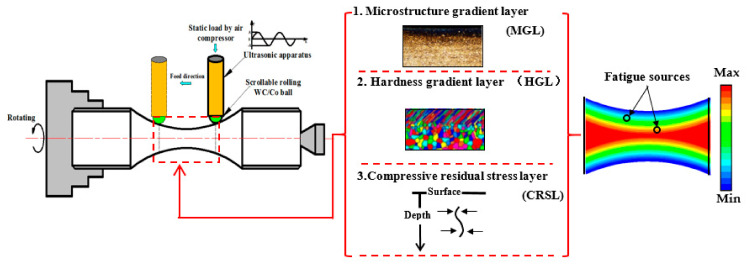
Fatigue source generation effect of multiple factors induced by the USRP (the colour gradient indicates the stress amplitude of the fatigue source).

According to GB/T3075-2008, each cylindrical specimen after heat treatment was processed into a standard fatigue specimen, as shown in Figure 1. The standard fatigue specimen was installed on the ultrasonic computerised numerical control lathe, and then the middle part of each specimen was mechanically polished along the axial direction. Then, the USRP surface modification treatment was performed. Different MGFs were formed to obtain different FCIPs so that the fatigue sources had different stress states, as shown in Figure 2. Based on the exploration of different FCIPs for fatigue specimens of the same size, it was difficult to induce a uniform FCIP on the smallest cross-section of the specimens of the same size. Therefore, this work used fatigue specimens of different sizes to explore this topic, and the corresponding USRP parameters are shown in Table 3. As shown in Table 3, the ϕ3.4 mm samples were treated by the USRP with three different parameters and were called U3.4-1, U3.4-2 and U3.4-3. In addition, the ϕ6.3 mm and ϕ8.5 mm samples were treated by the USRP with two different parameters and were called U6.3 and U8.5, respectively. Finally, a room temperature axial fatigue testing was carried out on five groups of specimens with different processes. The specifications of the fatigue test are as follows: QBG-200 type high-frequency fatigue machine, loading stress of 870 MPa, frequency 140 Hz and stress ratio of R = −1.

### 2.3. Experimental Measurement and Characterisation Method

A SUPPA40 Zeiss field emission scanning electron microscope (SEM, Zeiss, Analytik Jena city, Thuringia, Germany) was used to characterise the longitudinal section morphology of the surface and centre of the specimen after different processes. The microhardness values of the five processes along the depth direction were measured by an HVS-1000 microhardness tester (Lunjie Motor Instrument Company, Shanghai, China). The parameters of the microhardness tester are as follows: load of 100 g and holding pressure of 10 s. In addition, the microhardness value of each sample was repeatedly measured three times, the average value was taken, and the corresponding error range was saved. The axial residual stress values of the five processes along the depth direction were measured by a GNR type X-ray diffractometer (G.N.R. s.r.l.- Analytical Instrument Group, Milan, Italy), and the layer was stripped in situ on the worktable of an X-ray diffractometer by an electrolytic polishing machine. The parameters of the X-ray diffractometer are as follows: target material made of Cr-Kα, diffraction peak of {211}, scanning range of 147°–167°, diameter of incident tube of 0.5 mm and diffraction angles of ψ = 0°, ±10°, ±20°, ±30° and ±40°. In addition, the residual stress value of each sample was repeatedly measured three times, the average value was taken and the corresponding error range was saved. After the fatigue test, the morphology of the fatigue fracture surface and longitudinal section were observed with SEM. A layer of thin film was prepared in the fisheye area of two different FCIPs by the focused ion beam (FIB) technique with a Helios G4, and then the microstructure was observed under transmission electron microscopy (TEM) with an FEI Talos F200X (American FEI Company, Hillsboro, OR, USA). The preparation process was as follows: (1) Deposit a Pt protective layer in the target area; (2) etch the sample with gallium ions near the protective layer; (3) weld the nanoprobe and protective layer by spraying gallium ions; (4) bombard the bottom of the sample with a gallium ion beam to cut off the bottom of the connection between the sample and matrix; (5) while moving the nanoprobe, remove the sample, and then weld the sample on the copper mesh stag; finally, (6) further thin the sample with a gallium ion beam. The parameters of the FIB are as follows: voltage of 200 V to 30 kV, reduced beam intensity of 1 pA to 100 nA, electron beam resolution of 0.6 nm @ 15 kV and ion beam resolution of 2.5 nm at 30 kV. In addition, high-resolution transmission electron microscopy (HRTEM, American FEI Company, Hillsboro, OR, USA) was used to observe and analyse the relationship between the dislocation configuration and density of these two regions, as well as the interface structure and type of precipitates.

## 3. Results

### 3.1. Microstructure

**Figure 3:**
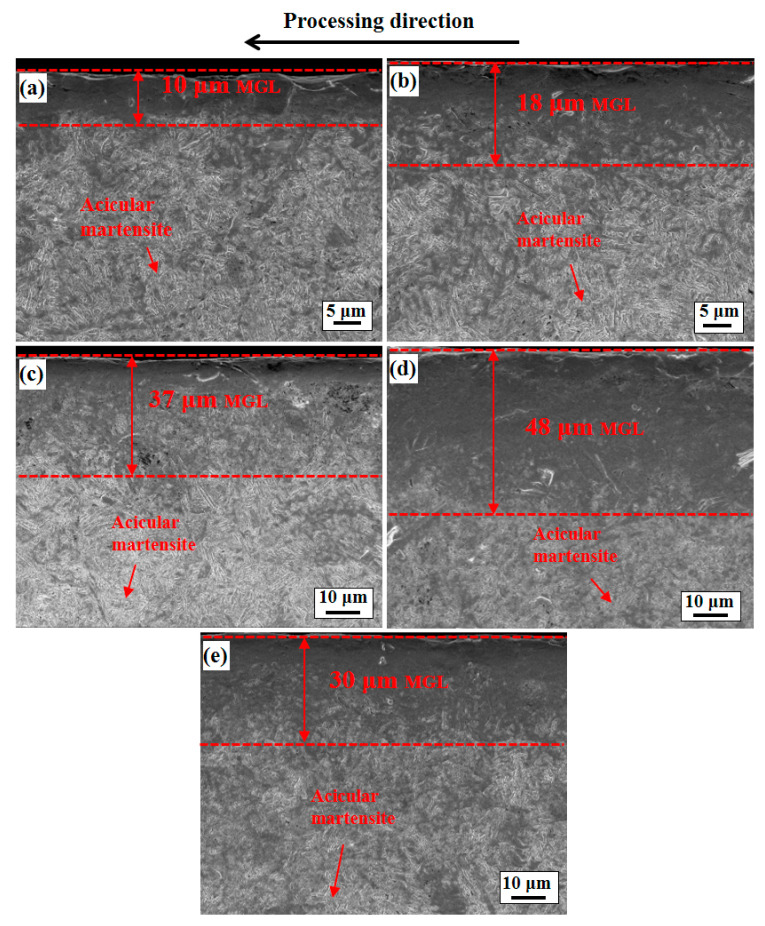
Longitudinal section SEM images of 51CrV4 spring steel specimens under five processes: (**a**) U3.4-1, (**b**) U3.4-2, (**c**) U3.4-3, (**d**) U6.3 and (**e**) U8.5.

SEM was used to observe the longitudinal section of the microstructure of the 51CrV4 spring steel specimens treated by different processes, as shown in Figure 3. After the USRP treatment, severe plastic deformation was observed on the surface of the five groups treated by different processes. With the difference in the USRP parameters, the deformation exhibited a gradient distribution along the thickness direction. The results show that the greatest thickness of the microstructure gradient layer (MGL) was approximately 48 μm (Figure 3d), whereas the thinnest MGL was approximately 10 μm (Figure 3a). The microstructure outside the MGL exhibited a typical acicular martensite structure. Ren et al. [18] observed that the microstructure of 50CrMnMoVNb spring steel modified with 3S was similar to that of 51CrV4 spring steel.

### 3.2. Hardness

**Figure 4:**
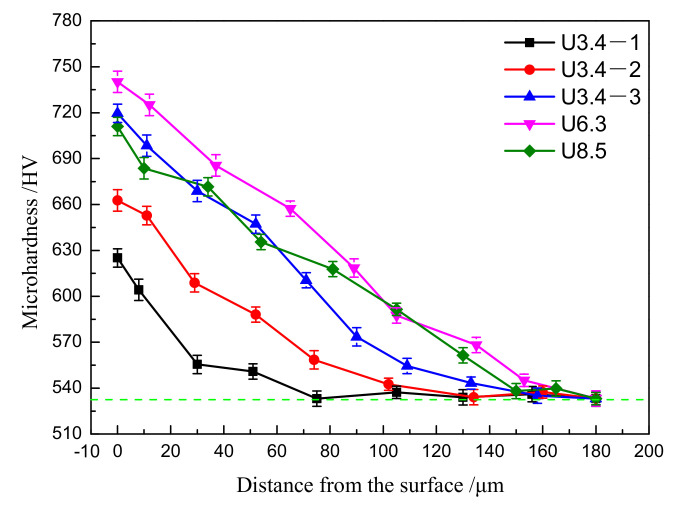
Microhardness of 51CrV4 spring steel specimens at the smallest diameter section under different treatment conditions.

Figure 4 shows the change in the microhardness gradient of 51CrV4 spring steel specimens under five processes. The surface microhardness under each process increases greatly, the surface microhardness of U6.3 is greatest (increasing from 530 ± 7 HV to 740 ± 6 HV, with an increase of 38.8%), and the work hardened layer is thickest (180 μm). In addition, each process exhibits a microhardness gradient distribution, and the maximum value appears on the outermost surface and then gradually decreases until it reaches a stable value. This is because after the material is processed by the USRP, the surface microstructure plastically deforms, which causes grain refinement and increases the dislocation density, thereby causing the surface layer of the material to undergo work hardening. This work hardening increases the surface hardness, which gradually decreases with increasing distance from the surface.

### 3.3. Residual Stress

**Figure 5:**
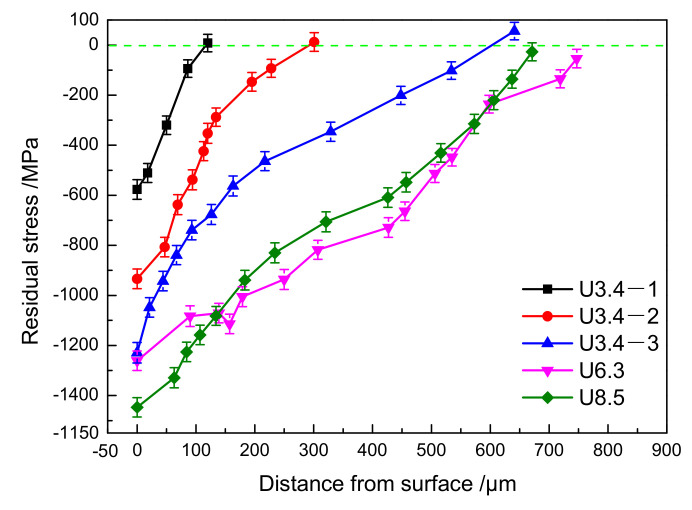
Compressive residual stress of 51CrV4 spring steel specimens under different treatment conditions.

Figure 5 shows the change in the residual stress gradient of 51CrV4 spring steel specimens with five processes. The surface of each process presents the compressive residual stress value and gradient distribution; the compressive residual stress value decreases with increasing distance from the surface. Under different processes with the same diameter (ϕ 3.4 mm), the spacing of the compressive residual stress gradient distribution is relatively obvious. The value of the compressive residual stress on the surface increases from 577 ± 29 MPa for U3.4-1 to 1229 ± 31 MPa for U3.4-3, and the depth of the CRSL increases from 120 μm for U3.4-1 to 650 μm for U3.4-3. However, under different processes with different diameters (ϕ3.4 mm, ϕ6.3 mm and ϕ8.5 mm), the compressive residual stress gradient distribution is relatively close. The value of the compressive residual stress on the surface of U8.5 is the largest (1447 ± 29 MPa), and the CRSL of U6.3 is the deepest (740 μm). In summary, the USRP can produce a higher residual stress gradient field on the surface of the material compared to that of conventional shot peening [21,22].

### 3.4. Fatigue Behaviour

**Figure 6:**
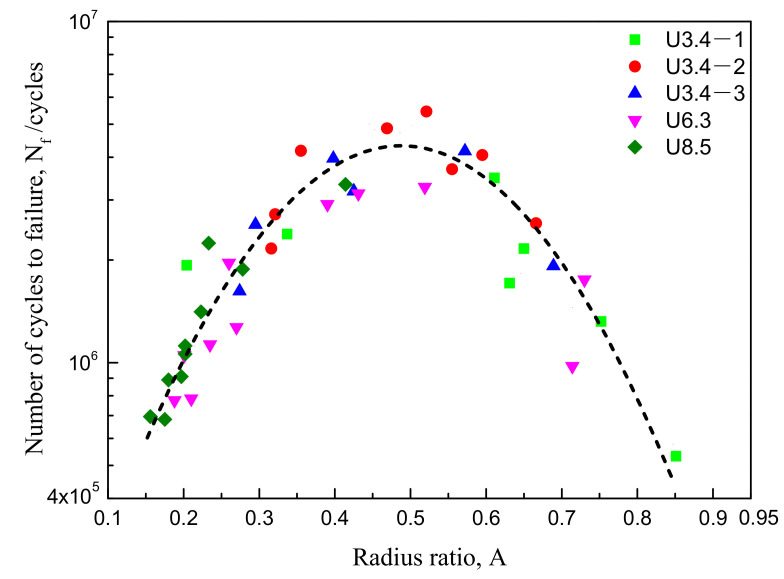
The relationship between *N_f_* and *A* for the axial fatigue test under the stress amplitude value of 870 MPa and different treatment conditions (to show the relationship between *N_f_* and *A* more clearly, the experimental data points are connected by dotted lines).

Wang et al. [23] believed that the crack initiation stage and the slow and intermittent small crack growth stages accounted for more than 95% of the fatigue life. In addition, the stress state of a small crack tip after fatigue crack initiation shows a gradient change. The closer an FCIP is to the centre of the specimen, the greater the stress triaxiality. Therefore, to reflect the stress state of the crack initiation position of fatigue specimens of different sizes, the ratio of the distance between the FCIP and the surface to the specimen radius is assumed to be the radius-ratio *A*. Thus, the larger the radius-ratio *A*, the closer the FCIP is to the centre, and the smaller the radius-ratio *A*, the closer the FCIP is to the surface. The relationship between fatigue life (*N_f_*) and radius-ratio (*A*) under the five processes with a stress amplitude of 870 MPa is shown in Figure 6. The fatigue life first exhibits an increasing tendency and then a decreasing tendency with the increase in the radius-ratio *A*. Thus, there is an optimal *A_C_*, corresponding to the highest fatigue life (*N_f max_*). When the value of *A* exceeds the value of *A_C_*, the fatigue life begins to decrease continuously; namely, the closer the FCIP is to the centre of the specimen, the lower the fatigue life. The value of *A_C_* at the peak of the parabola in the figure is approximately 0.5, and the largest fatigue life is 5.45 × 10^6^ cycles.

## 4. Discussion

The above analysis of the MGFs and fatigue behaviour of 51CrV4 spring steel indicates that the relationship between *A* and *N_f_* is based on the following behaviour: first, under the effect of the studied MGFs, the fatigue sources are distributed at different positions on the smallest cross-section of the specimen. If fatigue cracks initiate on the surface of the specimen, the fatigue crack tip maintains a plane stress state; if fatigue cracks initiate on the subsurface of the specimen, the fatigue crack tip maintains a plane strain state, and if fatigue cracks initiate at a centre far from the surface, the stress triaxiality of fatigue crack tip is higher. Therefore, with the different distances from the FCIP to the surface of the specimen, the stress state of the fatigue crack tip exhibits a gradient change. In addition, for the distribution law of stress triaxiality along the cross-section of the specimen, we can get inspiration from Bridgman’s principle [24]. This principle is that the stress triaxiality of the arc-notched specimen at the initial stage is

(1) $$\eta =\frac{1}{3}+\ln\left(1+\frac{\alpha^{2}-r^{2}}{2\alpha R}\right)$$

In the formula, *α* represents the radius of the smallest cross-section, *R* represents the radius of the notch, and *r* represents the distance to the centre of the smallest cross-section. Since this formula applies to the initial stage, no plastic deformation is generated, the arc-notched radius *R* is equal. Therefore, both *α* and *R* in formula (1) are constants, resulting in the stress triaxiality being a function of only *r*. To uniformly describe the distribution law of stress triaxiality on the cross-section of different sizes the specimens, the distance from the centre of the cross-section (*r*) is transformed into dimensionless, namely, *γ = r/α*, then *A = 1 − γ*, so the stress triaxiality of different FCIPs is

(2) $$\eta =\frac{1}{3}+\ln\left(1+\frac{\alpha [1-{\left(1-A\right)}^{2}]}{2R}\right)$$

Here, *η* increases with increasing *A*. When *A* = 1, namely, at the centre of the specimen, the stress triaxiality is the largest.

**Figure 7:**
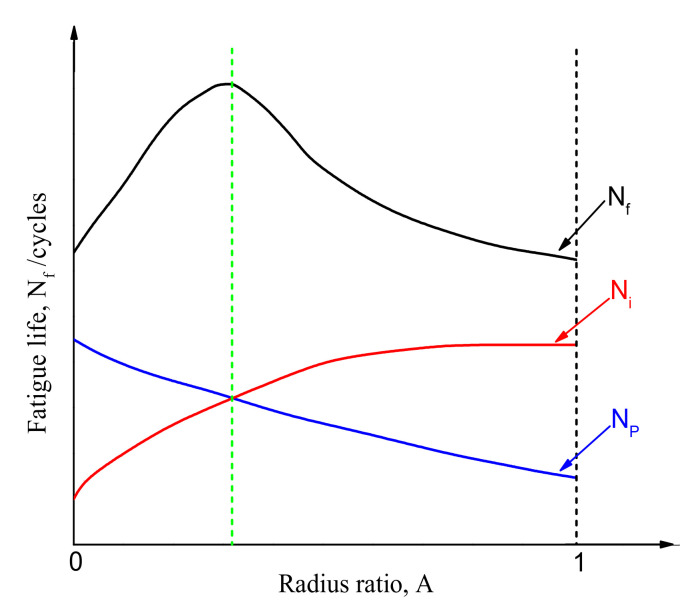
Relationship model between *N_f_* and *A*.

Second, the fatigue life (*N_f_*) is mainly composed of two parts: fatigue initiation life (*N_o_*) and fatigue propagation life (*N_p_*) [25]. When a FCIP shifts from the surface of specimen to the interior, according to the internal fatigue limit, the fatigue crack initiation resistance on the interior is greater than that on the surface [20]. This causes the crack initiation resistance of the interior to be greater than the crack growth rate, so *N_o_* increases, resulting in an increase in the fatigue life. However, when the FCIP further shifts towards the centre of the specimen, namely, *A* increases to exceed *A_C_*, the stress triaxiality at the fatigue crack tip gradually increases, which promotes a greater constraint on the front area of the small crack tip. As a result, the increase in stress concentration leads to the acceleration of the crack growth rate. This causes the crack growth rate to be greater than the crack initiation resistance, so *N_p_* decreases, resulting in a decrease in the fatigue life. Therefore, when the increasing range of *N_o_* is greater than the decreasing range of *N_p_*, the fatigue life increases; when the increasing range of *N_o_* is equal to the decreasing range of *N_p_*, the fatigue life reaches the maximum; when the increasing range of *N_o_* becomes less than the decreasing range of *N_p_*, the fatigue life begins to decrease. The relational model is shown in Figure 7. Therefore, the fatigue life first exhibits an increasing tendency and then a decreasing tendency with the increase in FCIP. Consequently, this article mainly analyses and verifies the MGF (MGL, hardness gradient layer (HGL), and CRSL) and the microstructural characteristics of different FCIPs (i.e., the fatigue fracture surface morphology, and the morphological characteristics of precipitates and dislocations). The specific analysis is summarised in the following section.

### 4.1. Major Factors Affecting the FCIP

When the FCIP is on the surface of the specimen, the surface roughness is an important influencing factor [26], but when the FCIP is in the interior of the specimen, the distribution of the MGF on the steel surface is an important factor inducing fatigue crack initiation [17]. Therefore, it is necessary to study the influence of the MGF on the FCIP.

#### Effect of the MGF on the FCIP

The CRSL can not only cause the closure effect during the crack growth process and reduce the growth rate, but also counteract a portion of the applied tensile stress, thus preventing fatigue crack initiation. In addition, MGLs and HGLs can prevent the formation of persistent slip bands (PSBs), thus delaying the growth of fatigue cracks [27,28,29]. Therefore, the MGF plays a decisive role in the FCIP. Table 4 summarises the comprehensive influence of the MGF on the distance between the FCIP and the surface under different processes. The table shows that the depths of the MGL and HGL for different processes are lower than the depth of the CRSL, so the influence of the depth of the CRSL on the FCIPs is mainly considered here. Under the depth of the CRSL of the same process, the FCIPs exhibits a significant dispersion, so that the fatigue life also exhibits a significant dispersion. The results show that there is no relationship between fatigue life and MGF. In addition, the depths of the CRSLs of all processes are all lower than the distance between the FCIP and the surface. This phenomenon follows the law of the influence of the CRSL on the FCIP, namely, the initiation of fatigue cracks is shifted to the tensile residual stress area [15,16]. In other words, the existence of the CRSL at the surface of the specimen can promote crack closure, thus reducing the crack propagation rate, namely, the corresponding effective stress intensity range is reduced (ΔK_eff_). When ΔK_eff_ is lower than the fatigue crack threshold (ΔK_th_), crack propagation does not occur, thus inhibiting crack initiation [30]. However, in the U3.4-3 and U6.3 processes, abnormal phenomena (the CRSL is deeper than the FCIPs) are observed for several FCIPs because although the distance between these fatigue sources and the surface is low, the corresponding values of compressive residual stress to FCIPs are close to the values of tensile residual stress (as shown in Figure 5). The FCIPs can also be interpreted to explain the initiation in the tensile residual stress area. Therefore, the function of the MGF is to distribute the fatigue sources at different positions on the smallest cross-section of the specimen, namely, to obtain different radius-ratio *A* values. Then the fatigue sources at different positions have different stress states, resulting in corresponding changes in the fatigue life.

### 4.2. Fractographic Analysis

**Figure 8:**
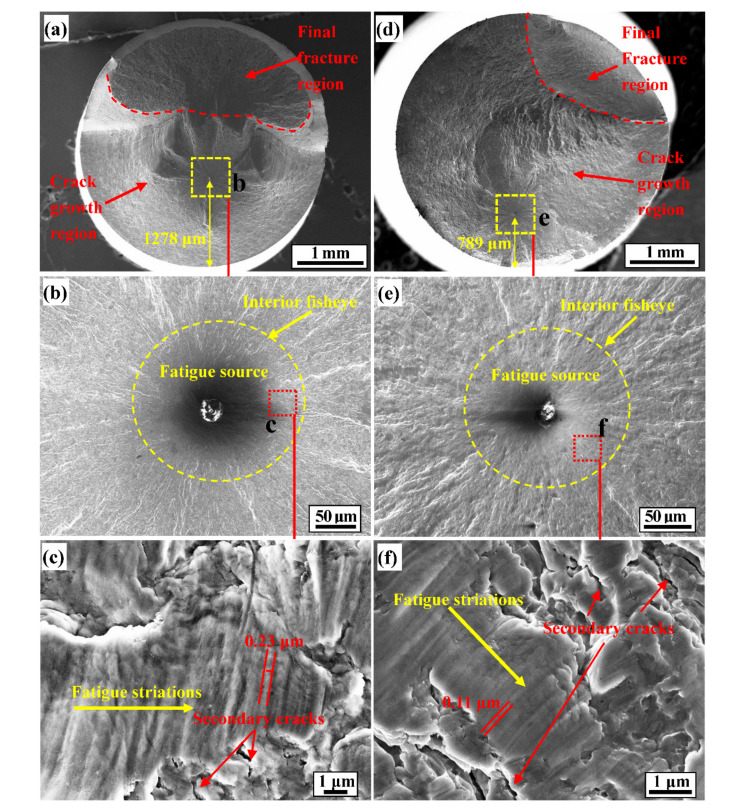
Fractographs of 51CrV4 spring steel with *A* = 0.752 (loading of 870 MPa and *N_f_* = 1.32 × 10^6^ cycles) and *A* = 0.469 (loading of 870 MPa and *N_f_* = 4.86 × 10^6^ cycles). (**a**) Macroscopic fracture for *A* = 0.752; (**b**) crack initiation area for *A* = 0.752; (**c**) fatigue striation features in fisheye area for *A* = 0.752; (**d**) macroscopic fracture for *A* = 0.469; (**e**) crack initiation area for *A* = 0.469; (**f**) fatigue striations features in fisheye area for *A* = 0.469.

**Figure 9:**
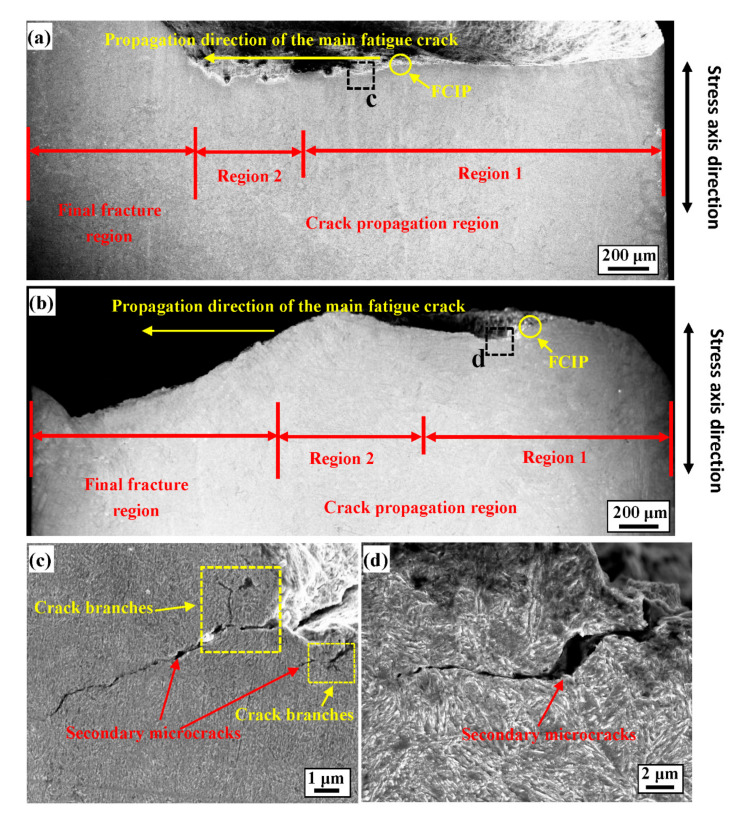
Longitudinal section morphology of the fatigue fracture surface with *A* = 0.752 (loading of 870 MPa and *N_f_* = 1.32 × 10^6^ cycles) and *A* = 0.469 (loading of 870 MPa and *N_f_* = 4.86 × 10^6^ cycles). (**a**) FCIP profile of macrocracks with *A* = 0.752; (**b**) FCIP profile of macrocracks with *A* = 0.469; (**c**) crack propagation characteristics in the short crack growth region for *A* = 0.752; (**d**) crack propagation characteristics in the short crack growth region for *A* = 0.469.

To understand the fatigue damage characteristics of spring steel, the fracture surfaces and their longitudinal section morphologies of fatigue specimens were analysed in detail. Figure 8 and Figure 9 show the fatigue fracture surfaces and their longitudinal section morphologies for *A* = 0.752 and *A* = 0.469. The purpose of this section was to study the fatigue crack initiation and growth mechanism of 51CrV4 spring steel and reveal the effect of different FCIPs on fatigue life.

#### 4.2.1. Fracture Surface Morphology Analysis

Figure 8 shows the fracture surface morphology of 51CrV4 spring steel with *A* = 0.752 after high-cycle loading (Figure 8a–c, loading of 870 MPa and *N_f_* = 1.32 × 10^6^ cycles) and *A* = 0.469 (Figure 8d–f, loading of 870 MPa and *N_f_* = 4.86 × 10^6^ cycles). Figure 8a,d shows the macroscopic fracture surface morphology of *A* = 0.752 and *A* = 0.469, respectively. The macroscopic fracture surfaces of the two states exhibit three regions: the fatigue initiation region, crack growth region and final fracture region [31,32]. In addition, the area of the stable crack propagation region at the fracture surface with *A* = 0.752 is smaller than that with *A* = 0.469. This is because when a FCIP is close to the centre of the specimen, the stress triaxiality increases, which makes the crack growth rate greater than the crack initiation resistance, which accelerates the growth of fatigue cracks and leads to a decrease in the area of the stable growth region.

Richards et al. [33] discovered that microvoids form in the stress triaxiality area at the fatigue microcrack tip and that the microvoids grow so that the ligament between the microvoids and the microcrack tip becomes thinner and eventually breaks, which leads to crack propagation and the formation of fatigue striations. The formation stage of microcracks is mainly concentrated in the interior of the fisheye area, so it is necessary to study the internal morphology of the fisheye area of fatigue fracture surface to confirm the relationship between the radius-ratio *A* and fatigue life. Figure 8b,c,e,f shows the magnified views of the fisheye area and fatigue striations inside the fisheye area in Figure 8a,d, respectively. Figure 8b,e shows that the FCIP of both fatigue fracture surfaces is induced by defects. Moreover, fisheye area size with *A* = 0.469 is smaller than that with *A* = 0.752, which is similar to the results obtained by Lei and Krewerth [34,35] after high-strength steel experiences very-high-cycle fatigue. In addition, Figure 8c,f shows that the number of secondary cracks in the fisheye area with *A* = 0.469 is greater than that with *A* = 0.752. The greater the number of secondary cracks, the more tortuous the path of the main crack propagation is, and the greater the frequency of deflection and the more energy absorbed, the slower the crack growth rate is, thereby increasing the fatigue life. In addition, it is evident that the larger the radius-ratio *A*, the wider the spacing of the fatigue striations is; the average fatigue striation spacings corresponding to *A* = 0.469 and *A* = 0.752 were measured to be 0.11 μm and 0.23 μm, respectively. The fatigue striation spacing is an indicator of the local fatigue crack propagation rate. Therefore, for quantitative analysis, the fatigue striations per unit length were measured, and then the crack growth rates were calculated [36]. The results of the average number of fatigue striations per micron in the internal fisheye area and the crack growth rate are shown in Table 5. This table shows that the crack propagation rates of *A* = 0.752 (0.22 μm/cycle) are higher than the crack growth rates of *A* = 0.469 (0.1 μm/cycle). As expected, the crack propagation rates increase with increasing fatigue striations.

The stress intensity factor (ΔK) is a physical quantity that describes the characteristics of the stress distribution in the crack tip area and directly reflects the strength of the elastic stress field at the crack tip. In addition, ΔK at the crack tip controls the crack growth rate, and the larger ΔK is, the faster the crack growth. Therefore, based on the theory of linear elastic fracture mechanics presented by Murakami et al. [37], the formula for calculating ΔK at the characteristic area of the internal fatigue fracture was proposed, namely

(3) $$\Delta K=0.5\sigma \sqrt{\pi \sqrt{area}}$$

In this formula, *σ* is the stress amplitude in MPa, and $\sqrt{area}$ is the size of the inclusion or the size of the fisheye area in μm. The calculated ΔK_fisheye_ for *A* = 0.752 is greater than that for *A* = 0.469 (the calculation results are shown in Table 5). In addition, in the stress field at the crack tip, McEvily [38] proposed that ΔK is related to the stress concentration factor K_t_, namely

(4) $$\Delta K=K_{t}\sqrt{\frac{\pi \rho_{e}}{4}}\sigma$$

In this formula, *ρ_e_* is the effective radius of the crack tip and *σ* is the far field stress. When the radius-ratio *A* exceeds *A_C_*, the stress triaxiality of a fatigue crack tip gradually increases, which promotes the enhancement of the elastic stress field in the front area of the crack tip; therefore, an increase in the stress concentration leads to a faster crack growth rate and reduced fatigue life.

#### 4.2.2. FCIP Profile Analysis

To verify the analysis results of the morphology of the fatigue fracture initiation area (the difference in the fatigue crack growth rate under different radius-ratios *A* of the two processes) and further reveal the different influence mechanisms of the two radius-ratios *A*, the cracks profiles near the FCIPs for the two radius-ratios *A* are analysed in detail. Figure 9 shows the characteristics of the fatigue fracture profile with *A* = 0.752 and *A* = 0.469. It can be observed that the two radius-ratios *A* divide the fatigue crack growth area into two regions along the main crack propagation direction (the crack propagation direction was from right to left): short crack growth (including the initiation region) (region 1) and long crack growth regions (region 2). Short crack growth has been found to account for more than 70% of the fatigue life of a component [25] and therefore could effectively reflect the effect of FCIP on fatigue life. Figure 9a,b shows that the crack propagation path of the short crack propagation area with *A* = 0.469 is more tortuous than that with *A* = 0.752. Therefore, as the crack propagation rate decreases, the tortuosity of the crack propagation path with *A* = 0.469 increases. In addition, Lütjering et al. [39] believed that a tortuous crack growth path would consume more energy than a smooth crack growth path and would have a positive contribution to the crack resistance, thus indicating that *A* = 0.469 has a higher fatigue life. Once the fatigue crack initiates, the stress distribution is no longer homogeneous, and stress concentrations develop near the crack tip [40]. During the crack propagation process, due to the stress concentration at the crack tip, many secondary microcracks are generated near the main crack path, which have different contributions to the hindrance and promotion of the main crack. Therefore, the two different short crack growth behaviours can be further verified by analysing the characteristics of the secondary microcracks near the fatigue source area below the main fatigue crack. As shown in Figure 9c,d, because the FCIP of *A* = 0.752 is close to the centre of the specimen, it is subject to greater stress triaxiality, which promotes greater constraints on the front area of the microcrack tip after fatigue crack initiation. Therefore, the propagation resistance in the front area of the microcrack is relatively large, and the microcracks have branches and continue to expand in the direction of least resistance, resulting in an increase in the crack length and a change in the propagation path. However, the microcrack path with *A* = 0.469 is rough, and the rough crack path promotes the closure of the crack tip, which further reduces the crack growth rate and improves the crack initiation resistance of the material as well as the fatigue life.

### 4.3. Dislocation Structures and Precipitate Characteristics near the FCIPs

**Figure 10:**
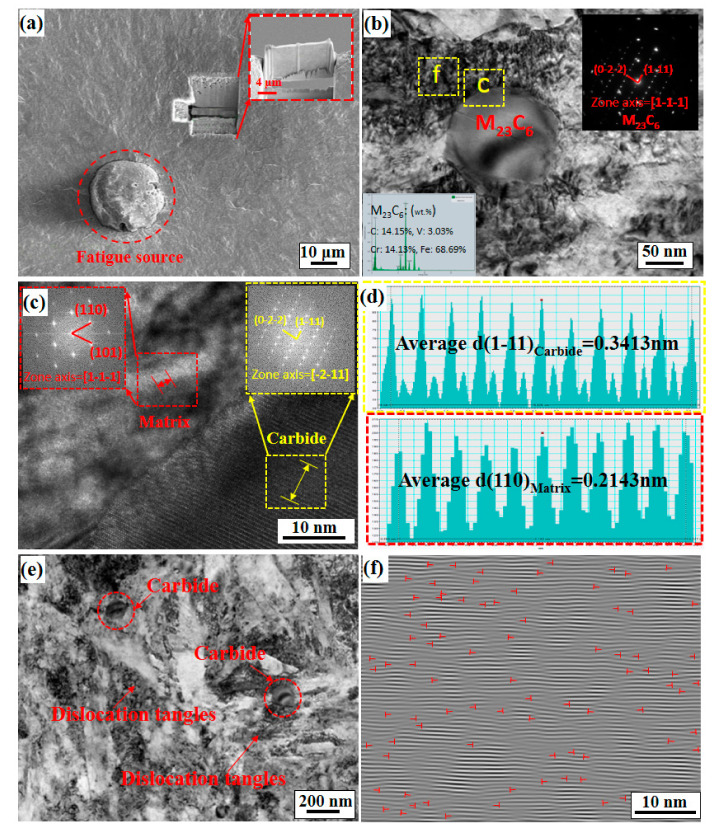
Microstructure image of the results of the axial fatigue test (σ = 870 MPa, *A* = 0.521 and *N_f_* = 5.45 × 10^6^ cycles): (**a**) sampling location of SEM-FIB; (**b**) TEM image of M_23_C_6_ carbides; (**c**) HRTEM images and the corresponding Fourier spot for region c in (**b**), suggesting that the relationship between the M_23_C_6_ precipitate and the matrix follows the [-2-11]_M23C6_//[1-1-1]_bcc-Fe_ orientation relationship; (**d**) the interplanar crystal spacing of the (1-11)_M23C6_ phase and (110)_α__-Fe_, respectively; (**e**) TEM image of the microstructure of the fisheye area; and (**f**) the corresponding FFT pattern for region f in (**b**).

**Figure 11:**
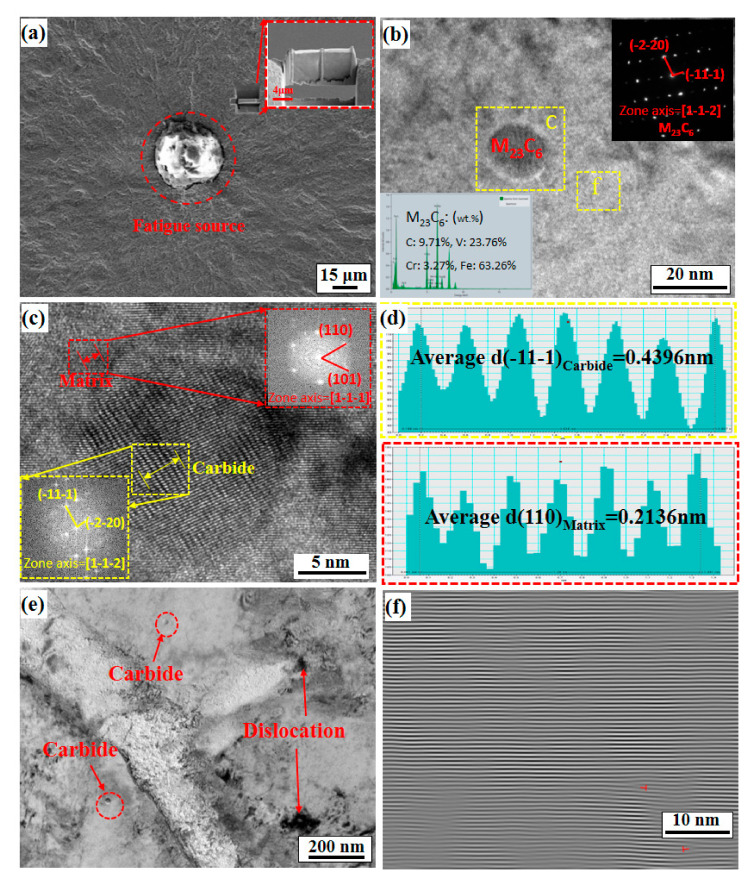
Microstructure image of the results of the axial fatigue test (σ = 870 MPa, *A* = 0.851, and *N_f_* = 5.32 × 10^5^ cycles): (**a**) sampling location of SEM-FIB; (**b**) TEM image of M_23_C_6_ carbides; (**c**) HRTEM images and the corresponding Fourier spot for region c in (**b**), suggesting that the relationship between the M_23_C_6_ precipitate and the matrix follows the [1-1-2]_M23C6_//[1-1-1]_bcc-Fe_ orientation relationship; (**d**) the interplanar crystal spacing of the (-11-1)_M23C6_ phase and (110)_α__-Fe_, respectively; (**e**) TEM image of the microstructure of the fisheye area; and (**f**) the corresponding FFT pattern for region f in (**b**).

In the previous section, the fatigue fracture morphology and the profile behaviour characteristics of the crack initiation area were analysed, and the behaviour characteristics of the microcrack growth for different radius-ratios *A* are initially determined. However, for the micromechanism of microcrack propagation resistance, further analysis of the dislocation structure in the microcrack tip area is needed. Figure 10 and Figure 11 show the micromorphologies for *A* = 0.521 and *A* = 0.851, respectively. A layer of TEM film is produced by FIB technology in the fisheye area of the two states (Figure 10a and Figure 11a) and then placed under TEM for observation and analysis. Figure 10b and Figure 11b show TEM microscopic images of carbides and their adjacent areas in the two states, and the energy-dispersive X-ray spectroscopy (EDS) and selected area electron diffraction (SAED) results in the inset show that both of the corresponding states precipitated as M_23_C_6_ carbide types. A similar carbide structure in martensitic steel was also reported by Refs. [41,42]. Second, Figure 10c,d shows that the M_23_C_6_ precipitate phase and the matrix follow the orientation relationship of [-2-11]_M23C6_//[1-1-1]_bcc-Fe_, where the spacing of the neighbouring (1-11)_M23C6_ planes is 0.3413 nm while that of the neighbouring (110)_α-Fe_ planes is 0.2143 nm, so the precipitate phase for *A* = 0.521 maintains an incoherent relationship with the matrix. However, Figure 11c,d shows that the M_23_C_6_ precipitate phase and the matrix follow the orientation relationship of [1-1-2]_M23C6_//[1-1-1]_bcc-Fe_, where the spacing of the neighbouring (-11-1)_M23C6_ planes is 0.4396 nm, while that of the neighbouring (110)_α-Fe_ planes is 0.2136 nm, so the precipitate phase for *A* = 0.851 also maintains an incoherent relationship with the matrix. Similar to the results analysed by Ref. [41], the lattice mismatch between the precipitates with *A* = 0.851 and the matrix is greater than that with *A* = 0.521. Because the interaction of the stress–strain field and dislocations generated by the interface structure relationship between the precipitate and the matrix will affect the fatigue performance of the material [43,44], Figure 10e and Figure 11e show the relationship between the precipitate phases and dislocations for *A* = 0.521 and *A* = 0.851, respectively. It can be observed that the dislocation density for *A* = 0.521 is higher than that of *A* = 0.851. The inverse fast Fourier transform (IFFT) can be performed on the adjacent areas of the carbides in the two states, and then the dislocation density can be calculated to confirm. The dislocation density of *A* = 0.521 is 3.28 × 10^16^ m^−2^ (Figure 10f), and the dislocation density of *A* = 0.851 is 0.08 × 10^16^ m^−2^ (Figure 11f)). This is because the dislocations pass through the incoherent precipitate phase through the Orowan bypass mechanism, so that the pinning effect of the nanosized carbide precipitates on the mobile dislocations makes the dislocation structure more stable [45]. The stable dislocation structure generates new movable dislocations under the influence of a constant alternating load, which makes the dislocation density in the local area very high. The dislocations become tangled, forming dislocation tangles. The dislocation tangles suppress the initiation and propagation of fatigue cracks and thus lengthen the fatigue life. However, when an FCIP moves towards the centre, the stress triaxiality of the corresponding fatigue crack tip gradually increases, which causes the front area of the crack tip to experience greater constraints. This causes the shear stress value of the front area of the crack tip to become smaller and makes it difficult to promote dislocation appreciation and movement, thereby making the dislocation density in the local area very low, so the increase in stress concentration leads to a faster crack growth rate and results in a decrease in fatigue life.

## 5. Conclusions

In this work, the effect of different FCIPs on the fatigue life after the USRP produces different MGFs on the surface of 51CrV4 spring steel was studied. The conclusions are as follows:

The fatigue life of the tested 51CrV4 spring steel samples with different MGFs exhibits a tendency of increasing first and then decreasing with the increase in radius-ratio *A*. In other words, there was a peak characteristic in the curve of FCIP versus fatigue life.

The fatigue sources were distributed at different positions on the smallest cross-section of the specimen owing to the effect of the MGF. Moreover, almost all the FCIPs were outside the MGF. When a FCIP shifts from the subsurface of the specimen to the internal region, namely, the radius-ratio *A* becomes closer to *A_C_*, more secondary cracks will form near the fatigue source. The formation of secondary cracks will slow the crack growth rate, leading to increased fatigue life. In addition, the interaction of incoherent carbides near the fatigue source and dislocations will increase the dislocation density. Dislocations were tangled with each other, forming dislocation tangles, which prevented the initiation and growth of fatigue cracks and increased the fatigue life.

However, when a FCIP shifts from the internal region to close to the centre, namely, when the radius-ratio *A* increases to exceed *A_C_*, the stress triaxiality experienced by the fatigue source will gradually increase. The larger stress triaxiality not only increases ΔK of the front area of the crack tip, which leads to a faster crack growth rate, but also makes it difficult for the dislocations to move, resulting in an increase in stress concentration. Therefore, all the above results will lead to the decrease in fatigue life.

## Figures and Tables

**Table 1:** Chemical composition (wt.%) of the 51CrV4 spring steel before heat treatment.

Element	C	Si	Mn	S	P	Cr	Ni	Cu	V
wt.%	0.496	0.252	0.98	0.0095	0.0056	1.12	0.03	0.06	0.147

**Table 2:** Tensile properties of the 51CrV4 spring steel after heat treatment.

Tensile Strength σ_b_/MPa	Yield Strength σ_s_/MPa	Elongation δ/%	Section Shrinkage ψ/%
1707	1547	9.95	41.57

**Table 3:** Related USRP parameters of the five processes.

Parameters	U3.4-1	U3.4-2	U3.4-3	U6.3	U8.5
Static pressure (N)	398	862	663	663	862
Spindle speed (r/min)	180	180	180	180	280
Feed speed (mm/r)	0.1	0.1	0.1	0.1	0.1
Repeated rolling times	3	3	3	3	3
Amplitude (μm)	6	6	6	6	6

**Table 4:** Descriptions of the comprehensive influence of the MGFs on the distance between FCIPs and the surface under different processes.

Technologies Conditions	MGL (μm)	HGL (μm)	CRSL (μm)	The Distance between FCIP and the Surface (μm)
U3.4-1	10	75	120	346, 573, 1039, 1072, 1105, 1278, 1446
U3.4-2	18	110	320	538, 546, 603, 798, 887, 943, 1012, 1132
U3.4-3	37	155	650	466, 501, 676, 722, 973, 1172
U8.5	30	160	670	671, 744, 764, 836, 857, 860, 949, 989, 1183, 1758
U6.3	48	180	740	591, 634, 660, 745, 820, 851, 1230, 1359, 1636, 2247, 2300

**Table 5:** Details of the statistical analysis of fatigue fracture surfaces for two values of radius-ratio *A* (*A* = 0.752 and *A* = 0.469) showing average per micron fatigue striations of different states, and different results are obtained in the fisheye area (da/dN, $\sqrt{area}$ and ΔK_fisheye_).

Specimen Condition	Striations (μm)	da/dN (μm/Cycle)	$\mathrm{Fisheye}\;\mathrm{Size},\;(\sqrt{area}\;\mu m)$	ΔK_fisheye_, (MPa∙m^1/2^)
*A* = 0.752–870 MPa	4.3 ± 0.04	0.22 ± 0.02	190.28	10.64
*A* = 0.469–870 MPa	9.0 ± 0.09	0.1 ± 0.01	155.09	9.60

## Data Availability

The data presented in this study are available on request from the corresponding author.
